# Coping with Temperature at the Warm Edge – Patterns of Thermal Adaptation in the Microbial Eukaryote *Paramecium caudatum*

**DOI:** 10.1371/journal.pone.0030598

**Published:** 2012-03-09

**Authors:** Sascha Krenek, Thomas Petzoldt, Thomas U. Berendonk

**Affiliations:** 1 Institute of Hydrobiology, Technische Universität Dresden, Dresden, Germany; 2 Molecular Evolution and Animal Systematics, Institute of Biology, University of Leipzig, Leipzig, Germany; University of Zurich, Switzerland

## Abstract

**Background:**

Ectothermic organisms are thought to be severely affected by global warming since their physiological performance is directly dependent on temperature. Latitudinal and temporal variations in mean temperatures force ectotherms to adapt to these complex environmental conditions. Studies investigating current patterns of thermal adaptation among populations of different latitudes allow a prediction of the potential impact of prospective increases in environmental temperatures on their fitness.

**Methodology/Principal Findings:**

In this study, temperature reaction norms were ascertained among 18 genetically defined, natural clones of the microbial eukaryote *Paramecium caudatum*. These different clones have been isolated from 12 freshwater habitats along a latitudinal transect in Europe and from 3 tropical habitats (Indonesia). The sensitivity to increasing temperatures was estimated through the analysis of clone specific thermal tolerances and by relating those to current and predicted temperature data of their natural habitats.

All investigated European clones seem to be *thermal generalists* with a broad thermal tolerance and similar optimum temperatures. The weak or missing co-variation of thermal tolerance with latitude does not imply local adaptation to thermal gradients; it rather suggests adaptive phenotypic plasticity among the whole European subpopulation. The tested Indonesian clones appear to be locally adapted to the less variable, tropical temperature regime and show higher tolerance limits, but lower tolerance breadths.

**Conclusions/Significance:**

Due to the lack of local temperature adaptation within the European subpopulation, *P. caudatum* genotypes at the most southern edge of their geographic range seem to suffer from the predicted increase in magnitude and frequency of summer heat waves caused by climate change.

## Introduction

Temperature is one of the most important environmental factors determining a variety of ecosystem elements, e.g. species ecophysiology, abundance and distribution, as well as species diversity and population dynamics [1]–[4]. Due to the current climate change, scientists started to re-evaluate the impact of elevated temperatures on the ecology of species. Here, ectothermic organisms are of special interest as their physiological performance is highly dependent on environmental temperature.

To make predictions of organisms' and population responses to global warming, studies on genetic and phenotypic diversity over a species' geographic range are important. Such investigations can unveil patterns of evolutionary temperature adaptation to the current thermal heterogeneity on Earth by determining which ectotherms have a high acclimatisation capacity and which only occur at specific temperatures. Adaptive phenotypic plasticity, for instance, may cause a higher tolerance to changing thermal conditions [5], [6], while local temperature adaptation might be detrimental.

Several studies could show a co-variation of latitude and thermal tolerance (e.g. [7], [8]) suggesting that organisms are adapted to the mean temperatures of their environment, but others failed (e.g. [9], [10]). Climate change is supposed to affect both climate averages and variability [11] and it has been shown that the thermal tolerance of many organisms is proportional to the magnitude of variation they are exposed to [12]. Organisms are also expected to be adapted to the thermal heterogeneity of their particular environment. This thermal heterogeneity increases with latitude. Therefore, organisms from variable climates, such as the temperate zone, should evolve a broad thermal tolerance resulting in *thermal generalists*. In contrast, tropical ectotherms, experiencing less variation in temperature, should be selected for narrow thermal niches resulting in *thermal specialists* [13], [14]. Consequently, analysing thermal niches of different populations along a latitudinal transect is necessary to understand the process of adaptation to novel thermal environments.

It has been shown that populations and individuals at the edge of the species range may suffer the most from increasing temperatures, because they often live close to the limit of their species' physiological thermal tolerance [15]. Therefore, it is not only important to investigate the intraspecific variation in species' thermal tolerance, but also to consider populations from the margins of their current distribution range. Especially if one would expect a *thermal generalist* pattern for ubiquitous species, populations at the ‘warm edge’ (such as the tropics or subtropics) might be most at risk due to global warming (cf. [16], [17]).

Because of the anticipated increasing risk of more intense, more frequent and longer-lasting heat waves during summer [18], species heat resistances are of particular significance [15], [19], [20], [21]. Here, thermal safety margins as well as the maximum warming tolerance are suitable characters to qualitatively elucidate the impact of climate change effects across latitude on different populations. These indicators are based on an organism's thermal tolerance and its relation to the local temperature regime [17]. Studies investigating species' current thermal adaptation patterns with respect to present-day and future environmental temperatures therefore allow predictions of species' and population responses to elevated temperatures.

Beside these patterns of evolutionary temperature adaptation obviously related to climate change, many other patterns are important in thermal adaptation with respect to species evolution and ecology. For example, the *warmer is better* hypothesis [22], [23], [24], which predicts a positive correlation between an organism's optimal temperature and its maximum performance; or the *Jack-of-all-temperatures is a master of none* hypothesis [25], which assumes an evolutionary trade-off between the performance breadth and the maximal performance of an organism, are controversially discussed. These patterns are relevant in a climate change context, too, but only few investigators have experimentally tested these basic ideas of evolutionary temperature adaptation 26–29.

**Figure 1:**
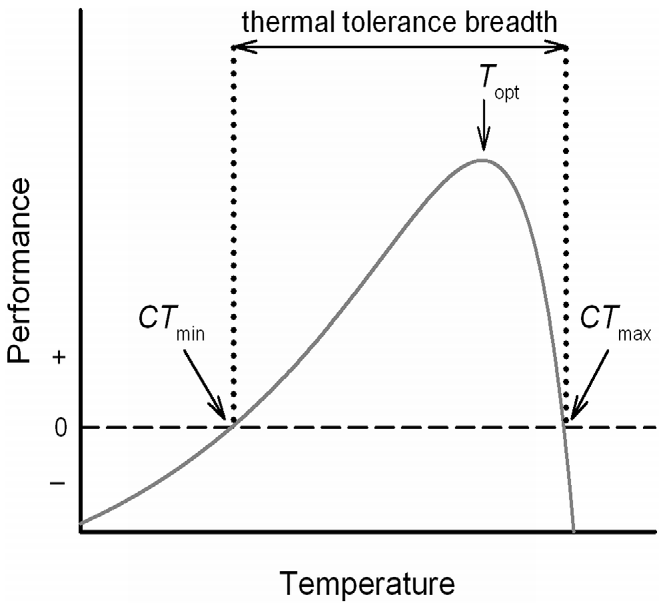
General shape of a thermal performance curve. Relationship between environmental temperature and a physiological rate of an ectotherm expressed as a thermal performance curve (grey line). The optimum temperature (*T*_opt_) specifies the temperature at maximum performance. The ecophysiological key characteristics critical thermal minimum (*CT*_min_) and maximum (*CT*_max_) delimit an organism's thermal tolerance.

For the investigation of such elementary hypotheses, the determination of thermal performance curves (Figure 1) provides a suitable framework to evaluate an organism's thermal tolerance [30]. Thermal performance curves (TPCs) allow estimations on how basic physiological functions are influenced by environmental temperature [17]. Furthermore, TPCs permit the calculation of ecophysiological key characteristics like the lower and upper critical thermal limits (*CT*_min_ and *CT*_max_) as well as the optimum temperature (*T*_opt_) and the maximum performance ([31]; cf. Figure 1]). Such key parameters are useful indicators for the thermal tolerance or thermal niche as well as for a potential environmental adaptation of different genotypes. As before mentioned, these ecophysiological characteristics can show a co-variation with latitude in metazoan species (e.g. [12], [32]), although other studies unveiled that the upper thermal limits of ectotherms vary little with latitude (e.g. [33], [34]). However, this has never been critically evaluated for microbial eukaryotes, which are not only important for aquatic ecosystems [35], [36], but also constitute well suited organisms for experimental evolution [37], [38].

While some recent studies have investigated the response of protozoan species to increasing temperatures (e.g. [39]–[42]), little is known about thermal adaptation patterns of globally distributed eukaryotic microbes and how temperature might affect the genetic diversity of natural populations. Furthermore, investigations on the intraspecific variation in species' thermal tolerance by considering populations from the margins of their current distribution range are rare as well.

In the present study, the microbial eukaryote *Paramecium caudatum* was used to investigate the intraspecific variation in temperature reaction norms of different genotypes. These were isolated from natural habitats along a latitudinal transect in Europe, while three genotypes from tropical habitats (Indonesia) served as a genetic and phenotypic outgroup. This globally distributed ciliate species inhabits the mud-water interface of littoral freshwater environments, which are considerably affected by atmospheric temperature changes [43], [44]. Consequently, *P. caudatum* has to cope with large temporal and spatial variations in temperature. It therefore constitutes a suitable ectotherm to test hypotheses in thermal adaptation as well as the consequences of climate change on such ubiquitous protists. Here, we performed temperature dependent growth experiments to (*i*) test for a hypothesized local temperature adaptation of different *P. caudatum* genotypes; (*ii*) investigate thermal constraints resulting from evolutionary temperature adaptation; and (*iii*) understand the sensitivity of *P. caudatum* to predicted future temperatures.

## Materials and Methods

### Sampling sites and Organisms

**Figure 2:**
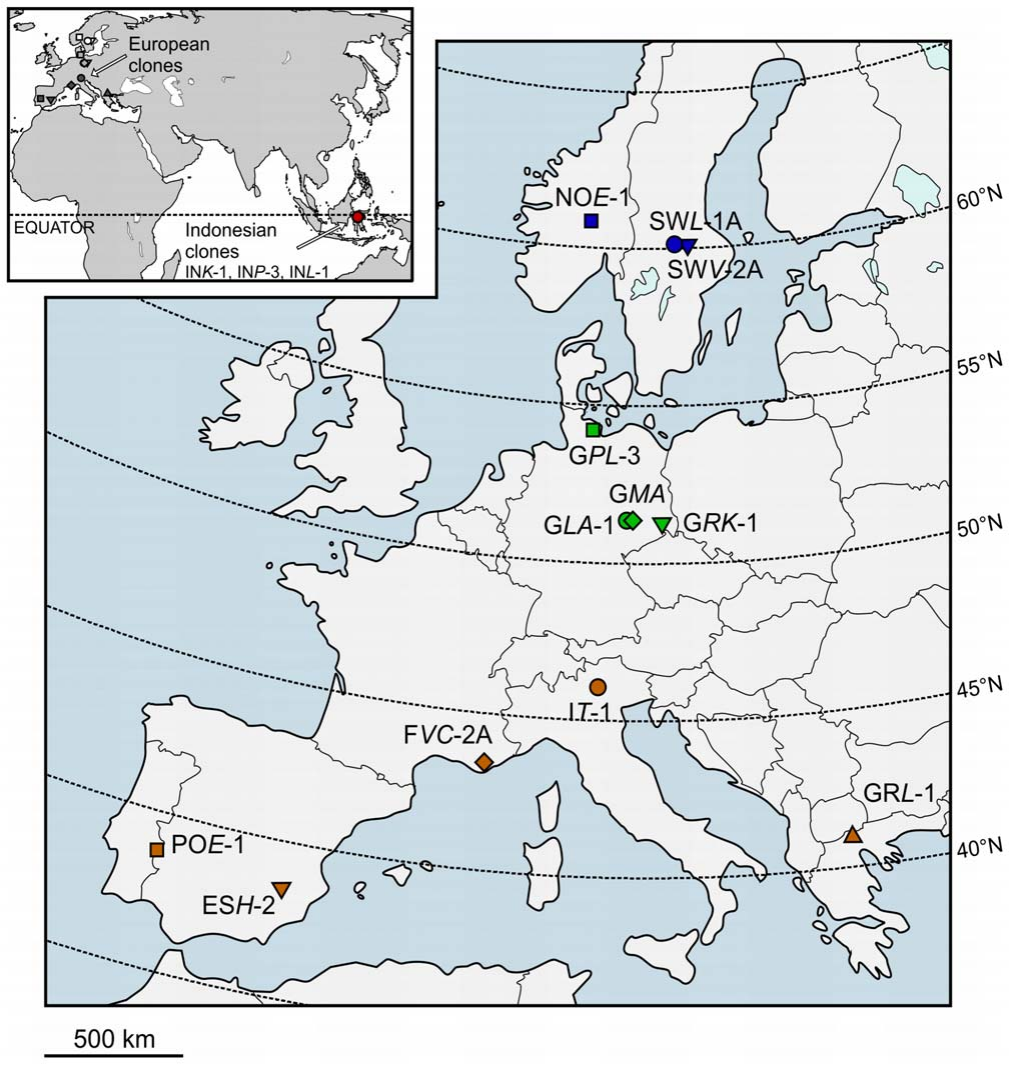
Geographic origin of investigated *Paramecium caudatum* populations. The small map shows the sampling points of all investigated *P. caudatum* clones within this study. The large map illustrates the sample sites within Europe in detail. Codes for clonal *P. caudatum* cultures refer to Table 1.

*Paramecium caudatum* cells were isolated from freshwater samples of 12 different natural habitats along a north-south transect in Europe as well as from three tropical habitats in Indonesia, Sulawesi (see Figure 2 and Table 1 for specifications). No specific permits were required for the described field studies. In Europe and Indonesia, work with *Paramecium* does not require specific permission and samples were not taken from water bodies where private property was indicated or from nature reserves where sampling is prohibited. The field studies did not involve endangered or protected species.

**Table 1:** Origin of *Paramecium caudatum* clones, genetic background and GenBank® accession numbers.

Clone Description	Place of Origin	Latitude	Longitude	Altidude	COI Haplotype*	Accession Number
NO*E*-1	Etnedal, Norway	60°51′42″N	9°41′17″E	539 m	PcCOI_a16	FN256274
SW*L*-1A	Ludvika, Sweden	60°7′43″N	15°10′10″E	177 m	PcCOI_a25	HQ149726
SW*V*-2A	Avesta (Norberg), Sweden	60°6′21″N	15°58′7″E	199 m	PcCOI_a26	AM407719
G*PL*-3	Ploen, Germany	54°14′8″N	10°25′6″E	47 m	PcCOI_a07	FN256269
G*LA*-1	Leipzig, Germany	51°22′14″N	12°19′15″E	102 m	PcCOI_a20	FN256279
G*MA*-1A	Machern, Germany	51°21′47″N	12°38′3″E	144 m	PcCOI_a01	HQ149717
G*MA*-1B	Machern, Germany	51°21′47″N	12°38′3″E	144 m	PcCOI_a01	HQ149718
G*MA*-2	Machern, Germany	51°21′47″N	12°38′3″E	144 m	PcCOI_a05	HQ149719
G*MA*-3	Machern, Germany	51°21′47″N	12°38′3″E	144 m	PcCOI_a03	HQ149720
G*RK*-1	Raeckelwitz, Germany	51°15′22″N	14°13′19″E	162 m	PcCOI_a06	FN256268
I*T*-1	Trent, Italy	46°4′13″N	11°7′18″E	196 m	PcCOI_a08	FN256270
F*VC*-2A	Vins-sur-Caramy, France	43°25′49″N	6°7′36″E	194 m	PcCOI_a30	HQ149716
GR*L*-1	Livadia, Greece	41°0′27″N	22°16′34″E	1181 m	PcCOI_a31	HQ149721
PO*E*-1	Elvas, Portugal	38°46′36″N	7°10′17″W	150 m	PcCOI_a28	HQ149725
ES*H*-2	Hellin, Spain	38°29′34″N	1°47′55″W	522 m	PcCOI_a27	AM407720
IN*P*-3	Palu, Indonesia	0°56′27″S	119°53′60″E	36 m	PcCOI_e02	HQ149724
IN*K*-1	Lake “Kalimpaa”, Indonesia	1°19′35″S	120°18′32″E	1660 m	PcCOI_e01	HQ149722
IN*L*-1	Lake “Lindu”, Indonesia	1°19′57″S	120°3′6″E	996 m	PcCOI_e03	HQ149723

* following the COI haplotype determination of Barth et al. (2006).

The food bacteria *Enterobacter aerogenes* were obtained from the American Type Culture Collection (ATCC 35028) and the kanamycin-resistant strain *Pseudomonas fluorescens* SBW25 EeZY-6KX [45] was acquired from the University of Oxford.

### *Paramecium* Stock MAINTENANCE

The investigated *P. caudatum* stock cultures were maintained in a 0.25% Cerophyl infusion, prepared according to the methods of Sonneborn [46] with minor modifications [31]. Isolated cells were separated in 1 ml of filtrated habitat water in 24-well tissue culture plates (TPP® AG) to establish clonal cultures. Afterwards, cells were washed and maintained at 22°C in a Cerophyl infusion inoculated with *Enterobacter aerogenes* to establish mass cultures. Later, cultures were kept at 10°C, lowering the growth and ageing of *P. caudatum*. Previous to the start of the experiments, monoxenic *P. caudatum* cultures were established at 22°C in a Cerophyl infusion with *Pseudomonas fluorescens* serving as the only food bacteria (for details see [31]).

### Temperature Dependent Growth Experiments

Cells from exponentially growing, monoxenic *P. caudatum* cultures were transferred to tissue culture flat tubes and acclimatised to experimental temperatures between 7°C and 35.5°C in steps of ±1.5 K d^−1^. Cultures were kept in exponential growth phase (500–1000 cells ml^−1^) during the acclimation period by doubling the culture volume with *Pseudomonas fluorescens* inoculated Cerophyl infusion (CMP; pH 7.0) as appropriate (1–5 ml per day). Due to the different acclimation phases from 22°C up to 35.5°C or down to 7°C, respectively, temperature-dependent experiments were conducted time-delayed. All experiments were performed in microprocessor-controlled, cooled incubators obtained from BINDER GmbH (Type KB 53).

Before the experimental start, acclimatised *P. caudatum* pre-cultures were adjusted to ∼250 cells ml^−1^ with CMP. Two millilitres of these starting cultures were added to each microcosm containing 18 ml CMP and resulting in an initial abundance of ∼25 cells ml^−1^. Growth experiments were run in triplicate in 60-ml tissue culture flasks with filter lids (TPP® AG) over two to eight days depending on the experimental growth temperature. The bacterial start density was regulated to a saturating prey level of about 2•10^8^ cells ml^−1^. If the *P. caudatum* pre-culture densities were below 250 cells ml^−1^ because of growth-limiting temperatures (e.g. 7°C or ≥34°C), initial cell abundance was adjusted to the highest possible cell number (≥10 cells ml^−1^).

*Paramecium* cell abundance was estimated by sampling 1 ml every nine to 41 hours depending on the experimental growth temperature. This resulted in five to eight samples per replicate. For precise counting, cells were fixed by the addition of Bouin's solution [47] to a final concentration of 1%. Cell numbers were enumerated microscopically by threefold counting 100 µl to 300 µl subsamples using a dark field stereoscopic microscope (Olympus GmbH). The population growth rate (*μ*, d^−1^) for each replicate and at each experimental temperature was calculated over the period of exponential increase using the slope of the linear regression of log_e_-transformed cell densities versus time (*t*).

### Intraspecific Differentiation

To identify and distinguish the individual, clonal cultures of natural *P. caudatum* genotypes from different geographic regions, the mitochondrial cytochrome *c* oxidase subunit I (COI) gene was sequenced following the protocol of Barth *et al.* [48]. Five cells from each stock culture were washed four times in sterile Eau de Volvic®and then incubated overnight with 100 µl of 10% Chelex®solution and 10 µl Proteinase K (10 mg ml^−1^) at 56°C. Afterwards, the mixture was boiled for 20 min and frozen at −20°C; the supernatant was used for subsequent PCR reactions. Each PCR reaction mix contained 10 µl of Chelex® extracted genomic DNA, 10 pmol of each primer, 1 U Taq-polymerase (SIGMA, Taufkirchen, Germany), 1×PCR buffer with 2 mM MgCl_2_ and 200 µM dNTPs in a total volume of 50 µl. PCR conditions were as follows: 5 min initial denaturation (95°C); 35 cycles of 1 min at 95°C, 1 min at 50°C and 45 s at 72°C; and a final extension step of 5 min (72°C). Using the primers CoxL11058 and CoxH10176 (see [48]), an 880-bp fragment of the mitochondrial COI gene was amplified. After purification with the Rapid PCR Purification System (Marligen Bioscience, Ijamsville, USA), PCR products were directly sequenced. Sequencing reactions were performed in both directions and analysed on an ABI 3100 Genetic Analyzer (Applied Biosystems).

### Thermal Performance Curves

The calculation of thermal performance curves (TPCs) was performed to describe the temperature dependent growth rate data of the individual *P. caudatum* clones and to determine clone specific key ecophysiological characteristics. TPCs have a common general shape with a gradual increase from a lower critical temperature (*CT*_min_) to a thermal optimum (*T*_opt_) where the investigated biological function reaches its maximum. With a further increase in temperature above *T*_opt_ the TPCs show a rapid decline towards a critical temperature maximum (*CT*_max_; Figure 1). It was shown that the nonlinear *Lactin-2* optimum function [49] can adequately describe the temperature – growth rate relationship of *Paramecium caudatum* resulting in typically skewed TPCs with a right-shift towards warmer temperatures [31].

The TPC estimation was done by fitting nonlinear mixed-effects models [50] simultaneously to the whole data set. The mixed-effects models were compared with AIC based model selection at three hierarchical levels; the whole data set with common fixed effects for all 18 clones (null models *nm0a* and *nm0b*, cf. Table 2), with separate fixed effects for the two regions, Europe and Indonesia (model *nm2*, cf. Table 2) and with separate fixed effects for the four regions northern, central, southern Europe and Indonesia (model *nm4*, cf. Table 2). In all cases, all four original parameters of the *Lactin-2* function (*ρ*, *T*_max_, *Δ* and *λ*) were used as fixed effects. In model *nm0a*, all four parameters were also used as random effects while for models *nm0b*, *nm2*, and *nm4* only *T*_max_, *Δ* and *λ* were used because of the high correlation between the parameters *ρ* and *λ* resulting in a low model convergence. The decision which of the two parameters had to be omitted for *nm2* and *nm4* was made by comparing the respective AIC values (not shown). Model *nm0b* is shown for comparison only (cf. Table 2).

**Table 2:** Comparison of nonlinear mixed-effects models with different levels of spatial aggregation.

Model	df	AIC	BIC	log Likelihood	Test	Likelihood Ratio	*p*-value
*nm0a*	15	19.87894	81.00913	5.06053			
*nm0b*	11	83.86464	128.69345	−30.93232	*nm0a* vs *nm0b*	71.9857	<0.0001
*nm2*	15	33.44298	94.57317	−1.72149	*nm0b* vs *nm2*	58.42166	<0.0001
*nm4*	23	187.12564	280.8586	−70.56282	*nm2* vs *nm4*	137.68266	<0.0001

The null models *nm0a* and *nm0b* were fitted with common fixed effects for all regions, model *nm2* with separate fixed effects for the tropical and the European region and *nm4* with separate fixed effects for northern, central, southern Europe and the tropical region. For fixed effects the complete set of parameters of the *Lactin-2* model (*ρ*, *T*_max_, *Δ* and *λ*; cf. Eq.1) was used in all cases. In model *nm0a*, all four parameters were also used as random effects, while for models *nm0b*, *nm2*, and *nm4* only *T*_max_, *Δ* and *λ* were used.

Then, the ecophysiological characteristics *CT*_min_ and *CT*_max_ were derived numerically as the intersection points of the resulting thermal performance curve with the temperature axis (*μ* = 0). The maximum growth rate (*μ*_max, cal_) was calculated analytically as the growth rate (*μ*) at *T*_opt_ using the *Lactin-2* function (Eq.1), while *T*_opt_ was calculated using its first derivative (Eq.2) as follows:
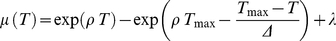
(1)

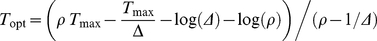
(2)where, the parameter *ρ* is a constant influencing *μ*_max_ and the slope of the low-temperature branch, *T*_max_ is the maximum temperature, and *Δ* defines the temperature range of the thermal inhibition above *T*_opt_. Parameter *λ* is an intercept parameter that forces the curve to intersect the abscissa at low temperatures and allows the estimation of *CT*_min_.

Finally, standard errors for both, the original *Lactin-2* function parameters (see Table S1) and the derived ecophysiological key parameters (Table 3) were estimated by nonparametric residual bootstrapping [51] with 1000 bootstrap replicates. For all further analyses based on these key ecophysiological characteristics, estimated data derived from the *nm0a* mixed-effects model fitting and bootstrapping procedure were used if not otherwise stated.

**Table 3:** Ecophysiological characteristics of individual *Paramecium caudatum* clones and the two regions, Europe and Indonesia.

Clone	*CT*_min_ (°C)	*T*_opt_ (°C)	*CT*_max_ (°C)	*μ*_max, obs_ (d^−1^)	*μ*_max, calc_ (d^−1^)	*TTB* (K)
NO*E*-1	0.90±1.43	29.01±0.16	32.25±0.02	2.34±0.03	2.14±0.04	31.35±1.45
SW*L*-1A	3.08±1.00	28.78±0.21	33.84±0.07	2.38±0.07	2.15±0.04	30.76±1.07
SW*V*-2A	−1.72±2.48	29.32±0.29	32.13±0.05	2.06±0.06	2.10±0.06	33.85±2.53
G*PL*-3	2.94±0.76	28.62±0.14	33.82±0.05	2.30±0.02	2.14±0.03	30.88±0.81
G*LA*-1	2.40±1.11	28.67±0.23	33.54±0.06	2.24±0.04	2.06±0.05	31.13±1.17
G*MA*-1A	4.00±1.12	28.52±0.20	33.33±0.06	2.47±0.05	2.13±0.04	29.33±1.18
G*MA*-1B	3.80±0.78	28.96±0.16	34.03±0.06	2.39±0.13	2.22±0.03	30.23±0.84
G*MA*-2	4.12±0.63	29.22±0.12	34.29±0.05	2.34±0.09	2.29±0.03	30.17±0.69
G*MA*-3	3.32±0.72	28.59±0.13	33.72±0.04	2.16±0.07	2.17±0.03	30.40±0.76
G*RK*-1	3.33±0.73	29.05±0.13	34.28±0.06	2.50±0.05	2.22±0.03	30.96±0.79
I*T*-1	3.14±2.76	29.00±0.39	33.02±0.11	1.99±0.03	2.04±0.09	29.88±2.87
F*VC*-2A	4.22±0.69	28.83±0.14	33.86±0.05	2.36±0.11	2.22±0.03	29.64±0.74
GR*L*-1	6.75±0.65	29.49±0.15	35.12±0.06	2.42±0.08	2.35±0.04	28.37±0.71
PO*E*-1	4.31±0.96	29.12±0.20	33.89±0.07	2.45±0.07	2.27±0.04	29.58±1.03
ES*H*-2	3.47±1.52	27.94±0.23	32.96±0.06	2.39±0.02	2.03±0.05	29.49±1.59
IN*P*-3	9.72±0.52	31.10±0.15	36.60±0.13	3.00±0.12	2.99±0.04	26.88±0.66
IN*K*-1	9.38±0.83	30.51±0.22	35.46±0.07	2.50±0.16	2.63±0.05	26.08±0.89
IN*L*-1	9.63±0.69	29.70±0.16	35.31±0.06	2.68±0.20	2.53±0.04	25.68±0.75
Europe	3.02±0.48	28.87±0.08	33.59±0.02	–	2.17±0.02	30.57±0.50
Indonesia	10.35±0.74	30.03±0.12	35.87±0.06	–	2.77±0.04	25.52±0.80

For each *P. caudatum* clone, the calculated critical minimum (*CT*_min_), maximum (*CT*_max_) and optimum temperatures (*T*_opt_) as well as the highest observed (*μ*_max, obs_) and calculated growth rates (*μ*_max, calc_) and thermal tolerance breadths (*TTB*) are reported as mean ± standard error of the mean.

### Climate Data

Investigating an organism's local temperature adaptation or its extinction risk due to climate change requires specific knowledge about the thermal conditions within its natural habitats. Here, we used site-specific temperature data to compare the clonal specific ecophysiological characteristics *T*_opt_ and *CT*_max_ with the current climate conditions of the specific habitats. Climate data were obtained from nearby meteorological stations (Table S2) or derived from the WorldClim database [52] using the program DIVA-GIS. In case of the meteorological station data, daily mean and maximum air temperature data of the years 2000–2011 (if available) were used to calculate the mean surface air temperature (*T*_hab, mean_) as well as the mean maximal surface air temperature (*T*_hab, max_), both for the warmest three months of the specific habitat. The WorldClim database is a set of interpolated global climate layers considering monthly precipitation as well as mean, minimum and maximum temperatures of the years ∼1950–2000. The database also provides 19 derived bioclimatic variables. The 2.5 arc-minutes resolution database was used to obtain the habitat mean temperature of the warmest quarter (bioclimatic variable 10 ≙ *T*_hab, mean_) and to calculate the habitat mean maximum temperature of the warmest three months (*T*_hab, max_). Furthermore, we used climate change data (2.5 arc-minutes) provided by DIVA-GIS to calculate future conditions for the respective habitats of the investigated *P. caudatum* clones. These data were derived from high-resolution simulations of global warming [53] using the CCM3 model and assuming a CO_2_ doubling until 2100 (see Table S2).

The use of such temperature data has proven controversial and it is well known that local and microhabitat temperature extremes and fluctuations can differ significantly from the regional average [54], [55]. However, it could be demonstrated that the summer lake surface water temperature of shallow lakes clearly correlates with the local air temperature [56]. Therefore, the use of local air temperature data seems to be a valid approach to estimate an aquatic ectotherm's performance temperature such as for *P. caudatum* that inhabits the littoral zone of freshwater environments.

### Statistical Analyses

Correlation analyses between each key ecophysiological characteristic (*CT*_min_, *T*_opt_, *CT*_max_) and the latitude of the respective natural habitats were performed to compare these thermal adaptation indicators with the geographical origin of the different clonal *P. caudatum* cultures. We used a subset of data containing all investigated European clones and a second dataset including also the Indonesian paramecia. Additionally, the latitudes of the European habitats were corrected for the altitude by assuming that 100 m elevation translates into a ∼100 km latitudinal increment within the temperate zone [57]. This correction was done to circumvent altitude effects on the thermal tolerance – latitude dependency. A correction for the Indonesian clones was disclaimed due to nonlinear and extraordinary steep elevational temperature gradients in the tropics [58]. The correlation analyses were performed with absolute values for the altitude-corrected latitude, where Spearman's correlation coefficient and the respective *p*-values were estimated by Pearson correlation on ranks.

Thermal safety margins (TSM = *T*_opt_−*T*_hab, mean_) as well as the warming tolerances of maximum temperatures (MWT = *CT*_max_−*T*_hab, max_) were calculated to correlate the clonal specific thermal adaptation indicators and the local climate conditions with the altitude-corrected latitudinal gradient (according to [17]).

A Spearman rank correlation analysis between all calculated maximum growth rates (*μ*_max, cal_) and *T*_opt_ values as well as all *μ*_max, cal_ data and thermal tolerance breadths (TTB = *CT*_max_−*CT*_min_) was used to test for the so-called *warmer is better* hypothesis [23] and the *Jack-of-all-temperatures is a master of none* hypothesis [25], respectively.

Additionally, the coefficients of variation (*CV*) of the mean growth rate of all investigated clonal *P. caudatum* cultures were analysed to estimate the intraspecific variation among all tested European clones as well as to assess intra-populational divergence. The calculation for each experimental temperature was as follows:
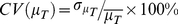
(3)where 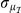 is the standard deviation of all growth rates (*μ_i_*) at the investigated temperatures (*T_i_*) and its arithmetic mean (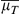).

To assess multivariate correlations between the genetic and the ecophysiological as well as geographic distances of the investigated *P. caudatum* clones, Mantel tests [59] were performed for the whole dataset as well as the European subset. Please note that this does not test for a causal link between the genetic variation of the COI gene and differing thermal tolerances as this gene was chosen to estimate the genetic differentiation within *P. caudatum*. It is a so called *barcoding gene* and facilitates comparisons of genetic variation within and among species [48], [60].

All statistical analyses were performed using the R system for statistical computing [61] with the add-on package nlme [62] for mixed-effects modelling and package vegan [63], [64] for Mantel tests.

## Results

### Intraspecific Variation in Thermal Performance

**Figure 3:**
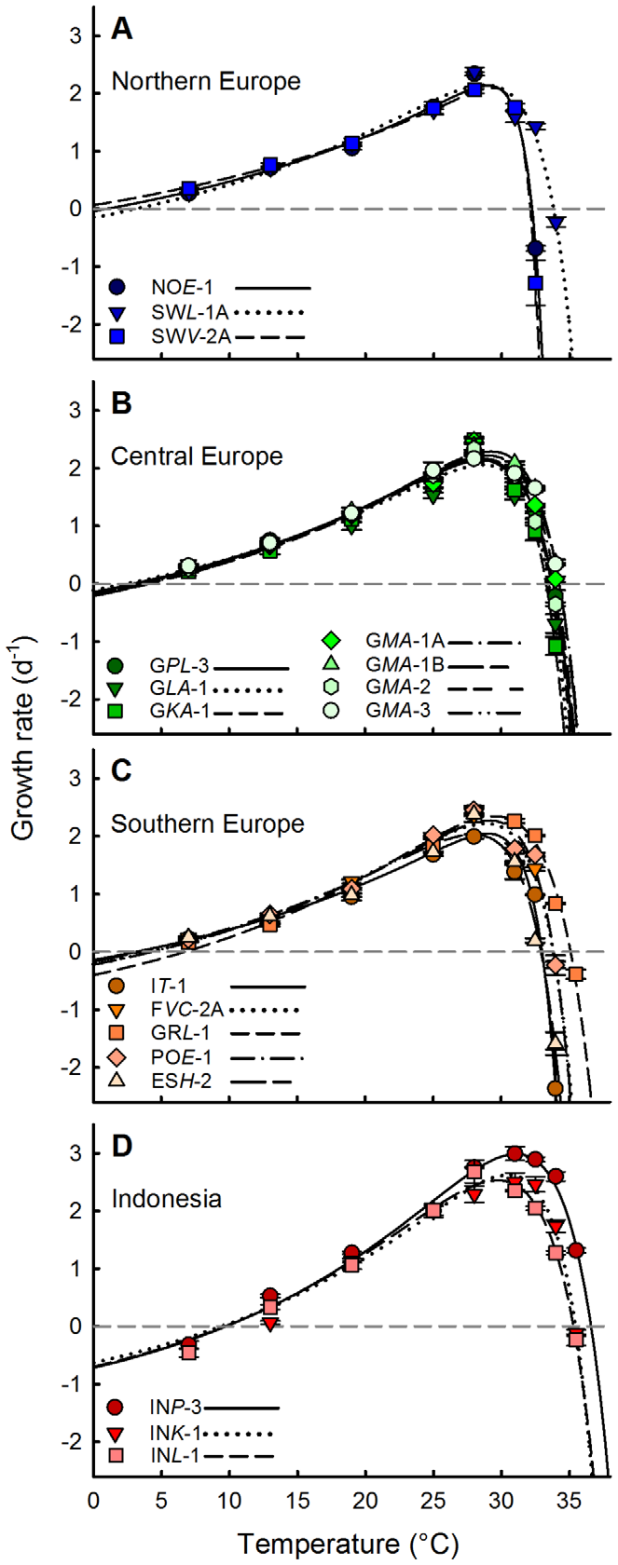
Thermal performance curves. Fitted thermal performance curves using the *Lactin-2* model to describe the growth rates – temperature relationship of all investigated clonal *P. caudatum* cultures. Clones were arranged according to their geographic origin: **A**) Northern Europe, **B**) Central Europe, **C**) Southern Europe and **D**) Indonesia. Symbols represent the mean ± standard error of the mean (n = 3) of the determined growth rates at the respective temperatures. Lines define the fitted thermal performance curves. Clonal descriptions refer to Table 1.

Using the *Lactin-2* model [49] to describe thermal performance curves (TPCs) of individual *Paramecium caudatum* clones resulted in typical left-skewed TPCs (Figure 3). These clonal specific TPCs allowed the calculation of ecophysiological key characteristics, which were qualitatively distinguishable between the different *P. caudatum* clones. For example, the Swedish clone SW*V*-2A possessed the lowest heat tolerance (*CT*_max_ = 32.13±0.05°C), while the clone from Greece (GR*L*-1) showed the highest (*CT*_max_ = 35.12±0.06°C) among all investigated European clones. Conversely, the Spanish clone (ES*H*-2) possessed a comparatively low heat tolerance (*CT*_max_ = 32.96±0.06°C) compared to another Swedish clone (*CT*_max, SW*L*-1A_ = 33.84±0.07°C). Not only the *CT*_max_ values showed high differences among the tested European clones, the calculated *CT*_min_ values were also considerably different (Δ*CT*_min, EU_ = 8.47±3.13°C, cf. Table 3). On the other hand, all European clones showed their highest growth rates (*μ*_max_) at the same experimental temperature of 28°C, while the calculated optimum temperatures (*T*_opt_) of the fitted TPCs ranged from 27.94±0.23°C (ES*H*-2) to 29.49±0.15°C (GR*L*-1).

The comparison between relative differences of intrinsic growth rate data of all European clones showed considerably larger differences with increasing distance from *T*_opt_. More precisely, large relative variations (*CV*) were obtained at low temperatures [*CV*(*μ*_7°C_) = 21.14%] and especially at temperatures above *T*_opt_ [*CV*(*μ*_32.5°C_) = 91.41%], compared to the low relative variation at *T*_opt_ [*CV*(*μ*_28°C_) = 7.50%]. While clones from the same habitat (G*MA*-1A, G*MA*-1B, G*MA*-2 and G*MA*-3) belonging to the same as well as to different COI haplotypes (see Table 1) showed seemingly similar reaction norms, we could detect some variation [*CV*(*μ*_7°C_) = 14.41%, *CV*(*μ*_28°C_) = 7.59%, *CV*(*μ*_32.5°C_) = 17.91%]. Performing one-way ANOVAs of clone specific growth rates at each experimental temperature suggests significant differences for the lowest and the highest temperatures tested (7°C, 32.5°C, 34°C; df = 3, *p*<0.001, with Bonferroni correction). This result indicates the existence of an intra-populational variation even though we found no significant differences for the intermediate temperatures.

While we could obviously detect only slight differences among the performance curves of different European *P. caudatum* clones (cf. Figure 3A–C), the Indonesian clones showed considerably different reaction norms compared to the European clones. For these paramecia we could identify significantly higher *CT*_min_, *T*_opt_, *CT*_max_ as well as *μ*_max_ values compared to the key characteristics of all European *P. caudatum* clones (Mann-Whitney U-test; U = 0, *p* = 0.002, n_1_ = 15, n_2_ = 3). In addition, an AIC based comparison of nonlinear mixed-effects models with different levels of spatial aggregation (Table 2) identified the model with separate fixed effects for the two regions, Europe and Indonesia (model *nm2*) as the second best model. While the null model *nm0a* with common fixed effects for all 18 individual clones was the most parsimonious and significantly superior model, the model with two regions was significantly better than the four regions model (*nm4*, cf. Table 2). This indicates a clear separation between the European and Indonesian clones, but not within the European paramecia.

### Correlation between Ecophysiology and Latitude

**Figure 4:**
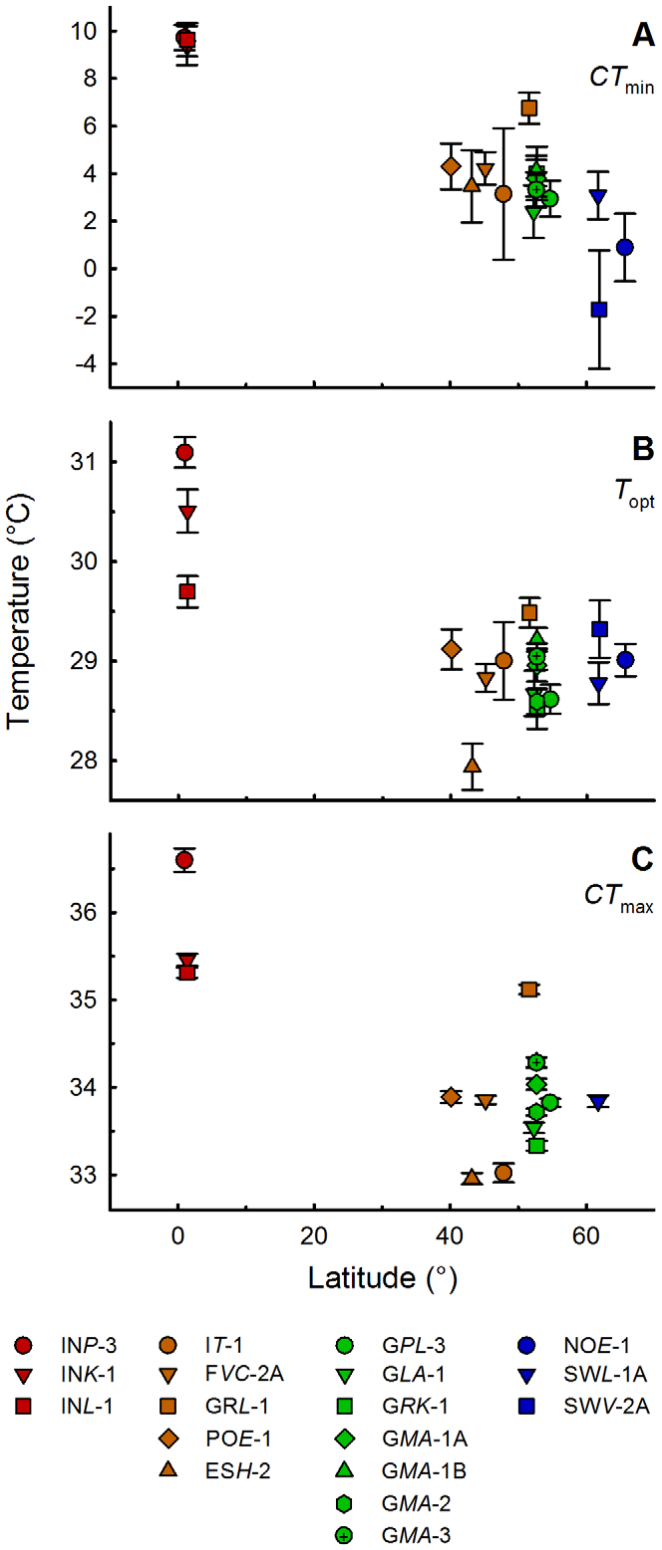
Latitude-dependent ecophysiology. Dependency between latitude and ecophysiological key characteristics: **A**) critical thermal minimum (*CT*_min_), **B**) thermal optimum (*T*_opt_) and **C**) critical thermal maximum (*CT*_max_). Symbols represent the mean ± standard error of the mean derived from the nonlinear mixed-effects model *nm0a* with residual bootstrapping. Latitudes of the European *P. caudatum* clones were corrected for altitude assuming that 100 m elevation translates into a 100 km latitudinal increment within the temperate zone. Spearman's rank correlation coefficients and the respective *p*-values are as follows for the whole dataset (n = 18): *CT*_min_ (*r_s_* = −0.795, *p*<0.001), *T*_opt_ (*r_s_* = −0.409, *p* = 0.092), *CT*_max_ (*r_s_* = −0.596, *p*<0.01); and for the European subset (n = 15): *CT*_min_ (*r_s_* = −0.647, *p*<0.01), *T*_opt_ (*r_s_* = 0.027, *p* = 0.924), *CT*_max_ (*r_s_* = −0.299, *p* = 0.279).

As illustrated in Figure 4, we obtained significant negative correlations for *CT*_min_ (*r_s_* = −0.795, *p*<0.001) and *CT*_max_ (*r_s_* = −0.596, *p*<0.01) with latitude using the complete dataset. When the correlation analyses were restricted to the European subset, we could only detect a significant correlation for *CT*_min_ (*r_s_* = −0.647; *p*<0.01; see Figure 4). These results were also supported by Mantel tests for the correlation between geographic and ecophysiological (*CT*_min_, *T*_opt_, *CT*_max_) distances. Here, analyses on the complete dataset revealed highly significant correlations for all tests (distance matrices of *CT*_min_, *T*_opt_ and *CT*_max_ vs. geographic distance matrix), while Mantel tests on the European subset showed non-significant relationships at all (cf. Table S3).

**Figure 5:**
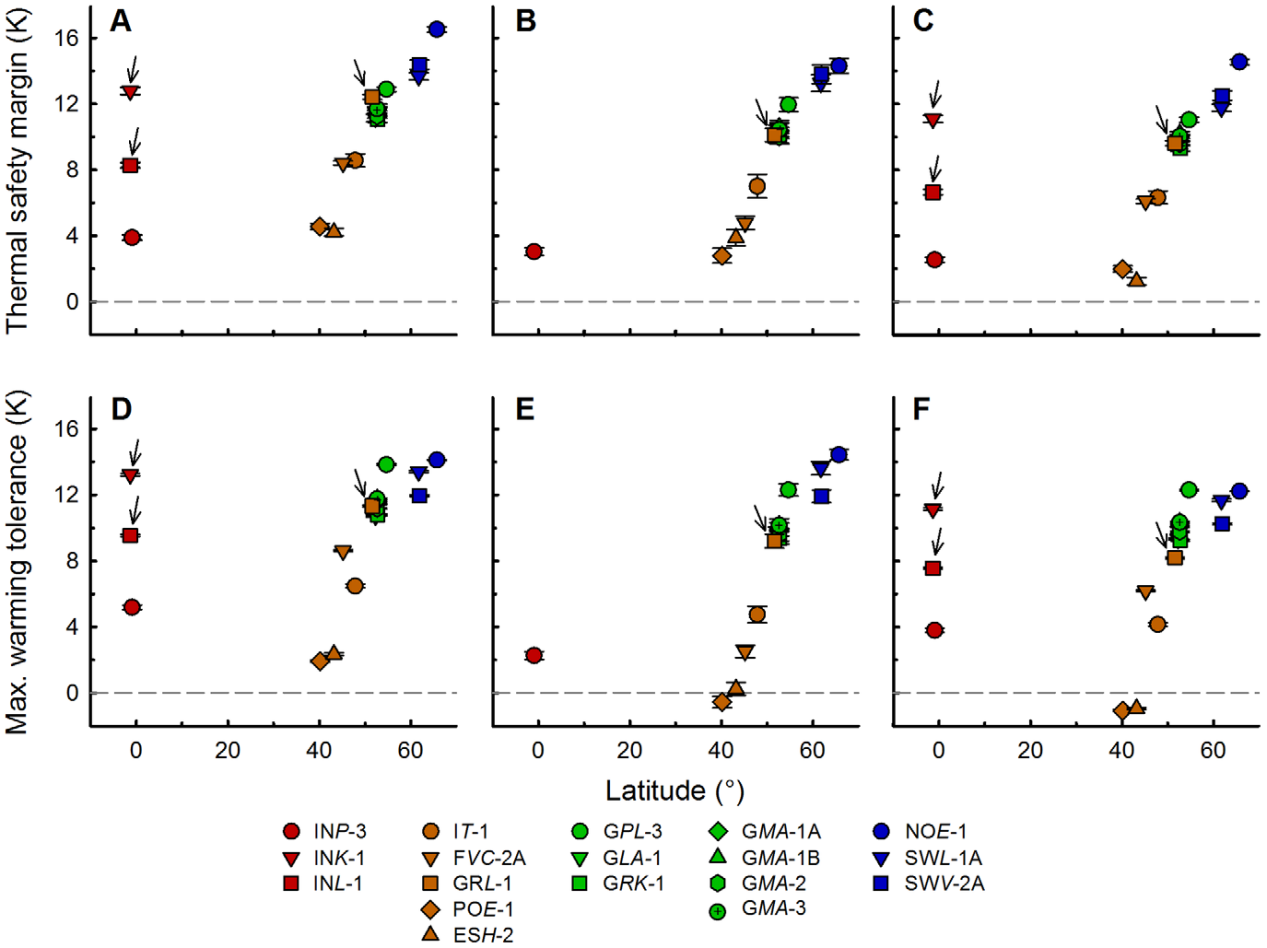
Latitudinal trends in thermal safety margin and maximum warming tolerance. Thermal safety margins (**A–C**) and maximum warming tolerances (**D–F**) of the investigated *Paramecium caudatum* clones were calculated using habitat temperatures from global climate layers (**A**+**D**), near-by meteorological station data (**B**+**E**) and from climate change projections (**C**+**F**). Symbols represent the mean ± standard error of the mean. Latitudes of the European clones were corrected for altitude assuming a 100 km increase in latitude for a 100 m increase in altitude. Arrows indicate *P. caudatum* clones from high altitude (cf. Table 1).

Our results further showed that thermal safety margins increased with latitude as well as altitude (Figure 5A–C). All low-latitudinal European paramecia from low altitude possessed considerably smaller thermal safety margins than all other clones tested. Interestingly, thermal safety margins derived from nearby weather stations were on average 1.36±0.65 K lower than data derived from the WorldClim database (cf. Figure 5A,B). This is potentially due to differently estimated time scales (years 2000–2011 versus ∼1950–2000) indicated by the fact that the first decade of the 21^st^ century apparently was the warmest since climate records began and by holding at least two summers most likely been the warmest in Europe since year 1500 [65], [66]. Taking this into account, the lowest observed thermal safety margin was 2.79±0.45 K for clone PO*E*-1 from Portugal and the highest for clone NO*E*-1 from Norway (16.51±0.16 K).

As shown in Figure 4C, we could not detect a significant decrease in *CT*_max_ with increasing latitude by analysing the European dataset. Therefore, the decrease in maximum air temperature with increasing latitude was considerably higher than the decrease in *CT*_max_ of the respective *P. caudatum* clones. This resulted in a steep increase in maximum warming tolerance with increasing latitude for the European *P. caudatum* clones (Figure 5D–F). Here, the low-latitude clones from Spain and Portugal showed the lowest tolerance window for extreme temperatures (Figure 5D). Analysing maximum warming tolerances derived from modelled climate change scenarios further revealed that these two low-latitudinal European clones (ES*H*-2, PO*E*-1) would show negative maximum warming tolerances (Figure 5F). The three investigated Indonesian clones, which served as a tropical outgroup, showed higher critical maximum temperatures than all tested European clones (Figure 4C). Hence, these clones showed positive maximum warming tolerances even for the predicted future maximum temperatures in their current habitats (Figure 5D–F).

### Thermal Constraints

**Figure 6:**
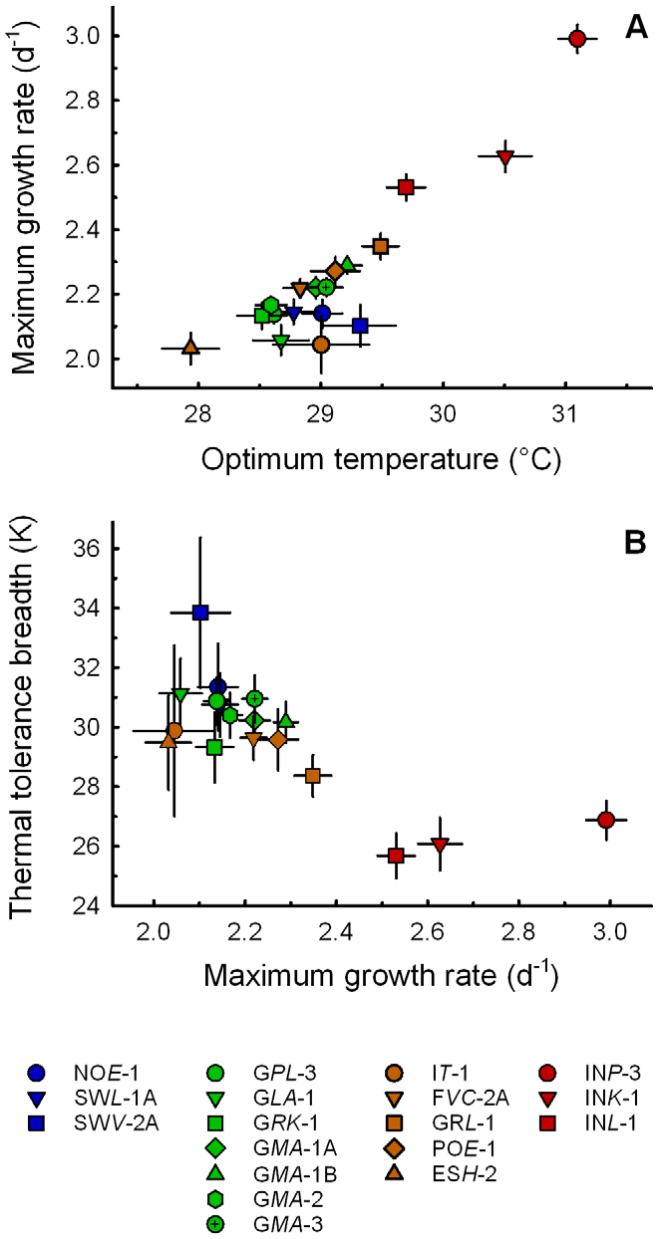
Thermal constraints. **A**) Relationship between calculated optimum temperature (*T*_opt_) and maximum growth rate (*μ*_max, cal_) for all investigated *P. caudatum* clones supporting *warmer is better*. Symbols represent the mean ± standard error of the mean derived from the nonlinear mixed-effects model *nm0a* with residual bootstrapping. Significance was tested with Spearman's rank correlation (n = 18, *r_s_* = 0.775, *p*<0.001). **B**) Trade-off between calculated maximum growth rate (*μ*_max, cal_) and thermal tolerance breadth (TTB = *CT*_max_−*CT*_min_). Symbols represent the mean ± standard error of the mean derived from the nonlinear mixed-effects model *nm0a* with residual bootstrapping. Significance was tested by using Spearman's rank correlation (n = 18, *r_s_* = −0,554, *p*<0.05).

Analyses of all optimum temperatures (*T*_opt_) and calculated maximum growth rates (*μ*_max, cal_) revealed a significant positive correlation of *T*_opt_ and *μ*_max, cal_ (n = 18, *r_s_* = 0.756, *p*<0.001; Figure 6A). This pattern was not significant among the European subset.

A negative correlation between the maximum performance (*μ*_max, cal_) and thermal tolerance breadth was detected using the complete dataset and performing Spearman's rank correlation (n = 18, *r_s_* = −0,546, *p*<0.05; Figure 6B). This indicates that higher maximum growth rates at higher optimum temperatures resulted in a narrower thermal tolerance (Indonesian clones), while a broader thermal tolerance was connected with lower maximum performance (European clones).

### Genetic and Ecophysiological Distances

Performing Mantel tests [59] on the complete dataset revealed highly significant correlations between ecophysiological (*CT*_min_, *T*_opt_, *CT*_max_) and genetic distances. Excluding the Indonesian clones from the analyses resulted in non-significant relationships (see Table 4). This indicates a missing correlation of the ecophysiology and the genetic distance within the investigated European *P. caudatum* clones using the mitochondrial COI gene as a phylogenetic marker and temperature dependent population growth rates as a fitness component. However, please note that the Indonesian clones possess both high geographical distances (10,715 km–13,261 km) as well as high genetic distances (0.073–0.087 substitutions per site) compared to the European *P. caudatum* clones, which exhibit comparatively low genetic distances among each other (0–0.02 substitutions per site). Performing Mantel tests for the genetic and geographic distances resulted in significant correlations for both datasets (Table S4).

**Table 4:** Mantel test for the correlation between genetic (*x* matrix) and ecophysiological distances (*y* matrix).

*y* matrix	*SSx*	*SSy*	*SPxy*	*Rxy*	*p*-value
whole dataset (n = 18)					
C*T*_min_	111932.2	1150.4	8710.8	0.768	0.001
*T*_opt_	111932.2	72.6	2071.5	0.727	0.003
C*T*_max_	111932.2	141.4	2492.4	0.627	0.001
European subset (n = 15)					
C*T*_min_	1240.8	341.4	113.3	0.174	0.196
*T*_opt_	1240.8	11.0	−16.1	−0.138	0.254
C*T*_max_	1240.8	46.2	15.1	0.063	0.324

*SSx* = sum of products of *x* matrix elements;

*SSy* = sum of products of *y* matrix elements;

*SPxy* = sum of cross products of corresponding elements of the *x* and *y* matrices;

*Rxy* = Mantel correlation coefficient.

## Discussion

### Latitude-dependent Ecophysiology

Generally, high-latitude *Paramecium caudatum* populations encounter lower temperatures than low-latitude populations, which should select for higher growth rates at low or high temperatures, respectively. Northern European *P. caudatum* clones should therefore exhibit lower critical minimum temperatures (*CT*_min_) to which the southern European clones were barely exposed to, while southern European clones should possess higher critical maximum temperatures (*CT*_max_). Our data partly support this general assumption of a co-variation of the critical thermal limits (*CT*_min_, *CT*_max_) and the optimum temperature (*T*_opt_) with the latitude when analysing the European dataset. Performing correlation analyses on this dataset revealed a significant dependence of *CT*_min_ with altitude-corrected latitudes, although we partly obtained high standard errors based on the used fitting procedure (cf. Figure 4A, Table 3). In addition, we could not detect such a correlation for *T*_opt_ and *CT*_max_ using the European dataset (Figure 4B,C). Our data therefore indicate a potential thermal adaptation of the northern European *P. caudatum* clones to lower winter temperatures only. These results are in accordance with a number of investigations on terrestrial ectotherms, which revealed that the lower critical temperatures significantly decline with increasing latitude while the upper thermal limits do not or are less variable [12], [33], [34], [67], [68]. This might be due to different costs for cold and warm tolerance, but up to now there is no clear evidence whether cold or warm adaptation came at a higher cost or if high heat tolerance is the ancestral state with low retaining costs (cf. [33], [69], [70], [71]).

When using the complete dataset that includes experimental data of all tested European as well as the three Indonesian clones, significant co-variations of *CT*_min_ as well as *CT*_max_ with latitude were obtained (Figure 4A,C). Additionally, the results of the model selection approach (Table 2) identified the model *nm2* with two regions (Europe and Indonesia) as significantly superior compared to the four regions model *nm4* (with regions for northern, central, southern Europe and Indonesia). These results imply a stronger impact of latitude on the thermal performance of the eukaryotic microbe *P. caudatum* when comparing data on a large inter-continental geographic scale, in contrast to the analyses on the intra-continental European scale. Further, significant positive correlations of all ecophysiological distances with genetic distances could be identified only when using the complete dataset, but not among the European subset (Table 4). Hence, the detected high genetic distances and significantly different ecophysiological characters of the Indonesian compared to the European clones as well as the results of the model selection approach suggest a large-scale biogeographic diversification within *Paramecium caudatum* on the phenotypic as well as the genetic level.

### Phenotypic Plasticity AND Thermal Adaptation

In general, the thermal performance of all investigated European *Paramecium caudatum* clones is indicative for a high phenotypic plasticity of this freshwater ciliate. Exemplified by the northern European clones, they showed a higher physiological optimum (around 29°C) compared to the temperatures they experienced in their natural habitats (Figure 5A). The optimum temperatures and the shapes of the thermal performance curves (TPCs) were reasonably similar for all genetically distinct clones from Europe (cf. Figure 3, 4B). Further, all European clones showed a general broad thermal tolerance and non-covarying *CT*_max_ as well as *T*_opt_ values with latitude (Figure 4B,C). These results disapprove the hypothesised latitudinal clines for thermal adaptation indicators such as heat tolerance and optimum temperature in European *P. caudatum* genotypes, but are in agreement with the *climatic variability* or the *seasonal variability* hypotheses [13], [72], [73]. These hypotheses claim that greater environmental variability at higher latitudes, for example due to seasonal changes, select for a more ‘generalist’ climatic tolerance and favours phenotypic plasticity within populations. Such a scenario would be also supported by the low genetic differentiation among the investigated European *P. caudatum* clones. This fact could be interpreted as an increased gene flow facilitated by the high phenotypic plasticity and the resulting low dispersal costs [13]. In that case, the high gene flow and high dispersal rates would also limit a potential local adaptation to specific habitat temperatures.

As our data revealed a comparatively broad thermal tolerance for all European clones with exceptional high *T*_opt_ and *CT*_max_ values, we would argue for an adaptive phenotypic plasticity among the investigated European populations resulting in *thermal generalists*. On the other hand, arguments for a potential thermal adaptation of the European *P. caudatum* to local microhabitat conditions are obvious due to the co-varying *CT*_min_ values with latitude (Figure 4A), but also because of large intraspecific growth rate variations at temperatures above *T*_opt_ (Figure 3). These differences at low and high temperatures indicate the existence of various ecotypes and a potential *microadaptation* to the local microclimate within the European *P. caudatum* clones. Temperature adaptation to microhabitat conditions has also been shown by several studies for ectothermic metazoans [74]–[77].

Along the three tested Indonesian clones, it was remarkable that in comparison to the European clones the averaged lower critical temperature of these tropical paramecia was shifted by more than +6 K, whereas *T*_opt_ and *CT*_max_ were only shifted by approx. +1.5 or +2.1 K, respectively. This means a reduction of their thermal tolerance breadth compared to the European *P. caudatum* clones. Given that in general the Indonesian clones were hardly ever stressed by temperatures below 10°C in their natural habitats, these tropical populations have not needed to adapt to lower temperatures. They have either lost the low-temperature tolerance or have never had this ability, depending on whether the tropical or the European clones represent the most ancestral phenotype. On the other hand, the Indonesian populations from low altitudes experience higher frequencies of hotter daily maximum temperatures. For example, the maximum air temperature for the natural habitat of clone IN*P*-3 could reach temperatures of up to 43°C. However, the mean maximum air temperature, which corresponds arguably better to the maximum water temperature because of the buffering capacity of water [78], is around 33°C. Here, we could show that the *CT*_max_ values of all investigated Indonesian *P. caudatum* clones (cf. Table 3) were somewhat beyond this temperature regardless of the elevation of their natural habitats. This result is indicative for a common thermal adaptation of the investigated Indonesian *P. caudatum* clones to the tropical temperature regime.

These findings support the general assumption that acclimatization and a high phenotypic plasticity is more likely in temperate species or populations at higher latitudes because the overall temperature variation increases with latitude. Thus, the evolution of broad thermal tolerances is needed that temperate organisms can cope with a large seasonal variation while organisms from less variable tropical environments should have evolved narrow thermal tolerances and reduced acclimation responses ([13], [32], [33]; but see [79]), as shown by our study. To generalise this pattern for *P. caudatum*, more clones from other tropical habitats need to be investigated.

### Thermal Constraints

In this study, a significant correlation between optimum temperature and maximum population growth rates could be shown (Figure 6A), which supports the so-called *warmer is better* hypothesis [22], [23], [24]. This *thermodynamic-constraint* hypothesis argues for a dependence of maximum performance on optimum temperature because of the thermodynamic properties of biochemical and physiological systems [24], [80]. Our results document that *P. caudatum* clones with higher optimum temperatures (*T*_opt_) have generally higher maximum growth rates (*μ*_max_) giving evidence for *warmer is better* concerning the growth performance as an important component of overall fitness. Nevertheless, we could not observe a significant correlation of *T*_opt_ and *μ*_max_ for the European subset, which indicates that the scale at which investigations are performed is of importance. Further studies on geographically well described organisms are necessary to understand if this finding is of general relevance for a variety of ectotherm species, for microbial eukaryotes only, or just for *Paramecium caudatum*.

The present study also provides an indication of an evolutionary trade-off between the performance breadth and maximum performance, which is known as the *Jack-of-all-temperatures is a master of none* hypothesis [25]. This result indicates that the selection for a broad thermal tolerance could result in a lower peak performance [81] while the selection for greater performance at a higher temperature would cause a correlated decrease in performance at lower temperatures [20]. Several studies provided mixed or no support of such specialist-generalist tradeoffs that constrain TPCs (e.g. [82]–[85]). Here, we could demonstrate such a negative correlation between maximum growth rates (*μ*_max_) and thermal tolerance breadths (Figure 6B). Especially the three tested Indonesian paramecia possessed higher *μ*_max_ values but narrower thermal tolerances than the European clones, mainly caused by a considerably larger shift of *CT*_min_ to higher temperatures compared to C*T*_max_. This result supports the above formulated suggestion that the European *P. caudatum* clones could be indicated as *thermal generalists* with a maximised performance breadth while the Indonesian clones seem to be *thermal specialists* with a maximised peak performance at higher temperatures.

### Sensitivity to Predicted Increasing Temperatures

The impact of the ongoing climate change on an ectotherm's fitness depends on numerous factors, including the community responses or the resource availability and the temperature-specific resource demand [86]. However, due to the expected temperature rise of up to 5.8°C by the year 2100 and the anticipated increase in diurnal variability of summer temperatures in the Northern Hemisphere [18], temperature is one of the most important factors which can drive shifts within the structure of natural populations [1], [2], [3]. Therefore, climate change effects are often predicted to give rise to species extinctions [82], [87]. The consequence of this temperature effect will considerably depend on the genotype specific *T*_opt_ and *CT*_max_ values relative to the mean and extreme habitat temperatures [88]. Thermal safety margins as well as maximum warming tolerances are suitable characters to reveal the potential extinction risk of organisms due to increasing temperatures [17].

In the present study, positive thermal safety margins reflect the temperature range at which the respective *P. caudatum* clone may benefit from a future warming of the habitat due to increasing growth rates. Negative values are rather a measure of potential risk. Our analysis revealed considerably smaller thermal safety margins for the low-latitudinal and -altitudinal European and tropical paramecia compared to all other *P. caudatum* clones tested (Figure 5A). However, all investigated clones currently live in environments that are on average cooler than their physiological thermal optimum. All tested clones seem to potentially benefit from future increasing temperatures (Figure 5C) at least initially.

Nevertheless, a second important key characteristic especially in consideration of the expected increasing intensity, frequency and duration of summer heat waves is the maximum warming tolerance. This character illustrates the average increase in maximum temperatures which *Paramecium* can tolerate before harmful growth conditions will be reached. Unexpectedly, analyses of the maximum warming tolerance showed that the European paramecia from low latitude and altitude currently experience near- or even above-lethal temperatures during summer (Figure 5E). Furthermore, the predicted future maximum temperatures of the low-latitudinal habitats (Portugal and Spain) are higher than their critical maximum temperatures (Figure 5F). Climate change models further predict the highest warming rates for low-latitude European habitats based on our dataset. Consequently, if these genotypes cannot adapt to the expected higher temperatures in their current natural habitats, they will potentially suffer from global warming. This seems not to be the case for the Indonesian and all high-latitudinal and –altitudinal European clones, which possess higher maximum warming tolerances (Figure 5D–F).

Our results, therefore, only partly support the hypothesis that tropical ectotherms are most at risk due to novel as well as disappearing climates in the tropics and subtropics in consequence of climate change [17], [89], [90]. In terms of the microbial eukaryote *Paramecium caudatum*, only the most southern European populations seem to be adversely affected by global warming. While some of the high-latitude European populations may actually benefit from increased temperatures by an enhanced population growth, the investigated Indonesian clones seem also not to suffer from the expected temperature rise. This is due to their adaptation to higher temperatures as well as the fact that temperature increase in the tropics is expected to be less intensive compared to temperate habitats [18]. However, tropical *P. caudatum* populations may become also affected by global warming, because the predicted changing environmental conditions such as temperature and precipitation seem to be comparatively heterogeneous across latitude [90], [91] and we have only a small dataset of tropical paramecia. Further, how climate change affects species abundances, distribution or diversity depends on the multigenerational response of their survival and reproduction within ecosystems [92]. The magnitude of temperature effects on species hinge on several factors such as food-web interactions, altered competition as well as the species specific acclimation or adaptation capacity [88], [93], [94]. Hence, experimental selection and micro-evolutionary studies as well as competition experiments of artificial populations pose an interesting research outline for future studies.

### Conclusions

All investigated European clones showed a broad thermal tolerance with high upper thermal limits as well as similar optimum temperatures, which is indicative for *thermal generalists*. These genetically closely related *P. caudatum* clones may have been selected to perform well in thermally variable environments utilising a high acclimation capacity. In contrast, the investigated Indonesian genotypes showed significantly higher optimum temperatures, maximum performances and critical thermal limits. This suggests local temperature adaptation of these tested clones to the less thermally variable tropical temperature regime. Further studies on additional ‘continental subpopulations’ of *P. caudatum* are needed to generalise the suggested *thermal generalist* and *thermal specialist* patterns in this ubiquitous microbial eukaryote.

Relating ecophysiological key characteristics such as optimum and maximum temperatures to current and predicted temperatures of all investigated *P. caudatum* clones suggest that only low-latitude European clones would be sensitive to global warming. During summer, they currently perform closer to their thermal limits than high-latitude European and tropical clones. Increasing temperature extremes, therefore, may severely affect their performance and fitness. Future investigations on experimental evolution would be useful to examine whether the different genotypes tested in this study can adapt to the predicted increase in temperatures and extreme events, or not.

## Supporting Information

Table S1 **Parameter estimates of the** ***Lactin-2*** **model.**
(DOC)

Table S2 **Geographical and meteorological details for natural habitats of investigated** ***Paramecium caudatum*** **clones.**
(DOC)

Table S3 **Mantel test for the correlation between geographic (** ***x*** **matrix) and ecophysiological distances (** ***y*** **matrix).**
(DOC)

Table S4 **Mantel test for the correlation between genetic (** ***x*** **matrix) and geographic distances (** ***y*** **matrix).**
(DOC)
