# Supplementary material for: Coping with Temperature at the Warm Edge – Patterns of Thermal Adaptation in the Microbial Eukaryote Paramecium caudatum
Source: PLoS One. 2012 Mar 9;7(3):e30598. doi: 10.1371/journal.pone.0030598 (PMC3302864; doi:10.1371/journal.pone.0030598)
Supplement: Table S1 — Parameter estimates of the Lactin-2 model. (DOC) [file pone.0030598.s001.doc]

**Table S1.** Parameter estimates of the *Lactin-2* model

| **Clone** | ***ρ*** | ***T*_max_** | ***Δ*** | ***λ*** |
| --- | --- | --- | --- | --- |
| NO*E*-1 | 0.0415 ± 0.0011 | 32.6195 ± 0.0311 | 1.1220 ± 0.0814 | -1.0380 ± 0.0517 |
| SW*L*-1A | 0.0452 ± 0.0010 | 34.5719 ± 0.0996 | 2.2918 ± 0.1743 | -1.1496 ± 0.0569 |
| SW*V*-2A | 0.0391 ± 0.0015 | 32.4165 ± 0.0382 | 0.8896 ± 0.1167 | -0.9351 ± 0.0820 |
| G*PL*-3 | 0.0456 ± 0.0008 | 34.5818 ± 0.0704 | 2.4017 ± 0.1216 | -1.1436 ± 0.0429 |
| G*LA*-1 | 0.0436 ± 0.0011 | 34.2317 ± 0.0863 | 2.1174 ± 0.1700 | -1.1105 ± 0.0616 |
| G*MA*-1A | 0.0459 ± 0.0010 | 34.0551 ± 0.0700 | 2.1563 ± 0.1360 | -1.2017 ± 0.0612 |
| G*MA*-1B | 0.0463 ± 0.0008 | 34.7738 ± 0.0950 | 2.3307 ± 0.1460 | -1.1928 ± 0.0466 |
| G*MA*-2 | 0.0469 ± 0.0007 | 35.0181 ± 0.0829 | 2.3362 ± 0.1225 | -1.2131 ± 0.0402 |
| G*MA*-3 | 0.0461 ± 0.0007 | 34.4645 ± 0.0579 | 2.3588 ± 0.1018 | -1.1654 ± 0.0399 |
| G*RK*-1 | 0.0461 ± 0.0008 | 35.0337 ± 0.0921 | 2.4285 ± 0.1355 | -1.1657 ± 0.0453 |
| I*T*-1 | 0.0424 ± 0.0022 | 33.5832 ± 0.0894 | 1.5807 ± 0.2010 | -1.1423 ± 0.1356 |
| F*VC*-2A | 0.0468 ± 0.0007 | 34.6026 ± 0.0701 | 2.3153 ± 0.1156 | -1.2182 ± 0.0410 |
| GR*L*-1 | 0.0502 ± 0.0010 | 36.0622 ± 0.0827 | 2.9244 ± 0.1528 | -1.4033 ± 0.0547 |
| PO*E*-1 | 0.0465 ± 0.0009 | 34.5752 ± 0.1005 | 2.1256 ± 0.1636 | -1.2220 ± 0.0566 |
| ES*H*-2 | 0.0456 ± 0.0014 | 33.7315 ± 0.0662 | 2.3017 ± 0.1528 | -1.1716 ± 0.0773 |
| IN*P*-3 | 0.0556 ± 0.0014 | 37.5006 ± 0.1975 | 2.9690 ± 0.2771 | -1.7162 ± 0.0683 |
| IN*K*-1 | 0.0519 ± 0.0014 | 36.2966 ± 0.1208 | 2.4513 ± 0.2295 | -1.6275 ± 0.0837 |
| IN*L*-1 | 0.0549 ± 0.0013 | 36.3725 ± 0.0951 | 3.1448 ± 0.1846 | -1.6957 ± 0.0713 |
| Europe | 0.0448 ± 0.0005 | 34.2556 ± 0.0288 | 2.0468 ± 0.0630 | -1.1451 ± 0.0260 |
| Indonesia | 0.1565 ± 0.0060 | 36.3214 ± 0.1229 | 6.1926 ± 0.1917 | -0.6107 ± 0.1137 |

Model parameters of the individual clones were estimated by fitting a nonlinear mixed-effects model without grouping (model *nm0a*) using the *Lactin-2* function. Parameter estimates for the two regions, Europe and Indonesia were derived from the grouping in model *nm2*. Standard errors were estimated by nonparametric residual bootstrapping.
