# Supplementary material for: Coping with Temperature at the Warm Edge – Patterns of Thermal Adaptation in the Microbial Eukaryote Paramecium caudatum
Source: PLoS One. 2012 Mar 9;7(3):e30598. doi: 10.1371/journal.pone.0030598 (PMC3302864; doi:10.1371/journal.pone.0030598)
Supplement: Table S2 — Geographical and meteorological details for natural habitats of investigated Paramecium caudatum clones. (DOC) [file pone.0030598.s002.doc]

**Table S2.** Geographical and meteorological details for natural habitats of investigated *Paramecium caudatum* clones

| **Clone** | **WorldClim Database** | | **Climate Change Model** | | **Weather Stations** | | | | | |
| --- | --- | --- | --- | --- | --- | --- | --- | --- | --- | --- |
|  | *T*_hab, mean_ | *T*_hab, max_ | *T*_hab, mean_ | *T*_hab, max_ | Name | Latitude | Longitude | Altitude | *T*_hab, mean_ | *T*_hab, max_ |
| NO*E*-1 | 12.50 | 18.13 | 14.47 | 20.03 | Vest-Torpa | 60°55'48"N | 10°1'48"E | 542 | 14.71 ± 0.28 | 17.82 ± 0.30 |
| SW*L*-1A | 15.10 | 20.43 | 17.02 | 22.17 | Skeppmora | 60°6'36"N | 15°6'36"E | 240 | 15.52 ± 0.29 | 20.23 ± 0.34 |
| SW*V*-2A | 14.95 | 20.20 | 16.82 | 21.87 | Skeppmora | 60°6'36"N | 15°6'36"E | 240 | 15.52 ± 0.29 | 20.23 ± 0.34 |
| G*PL*-3 | 15.73 | 20.00 | 17.58 | 21.53 | Doernick | 54°8'24"N | 10°20'60"E | 28 | 16.66 ± 0.27 | 21.53 ± 0.32 |
| G*LA*-1 | 17.53 | 22.80 | 19.20 | 24.20 | Leipzig-Schkeuditz | 51°24'45"N | 12°14'5"E | 144 | 18.51 ± 0.32 | 23.92 ± 0.38 |
| G*MA*-1A | 17.33 | 22.53 | 19.02 | 23.93 | Leipzig-Holzhausen | 51°19'2"N | 12°24'45"E | 148 | 18.61 ± 0.29 | 24.11 ± 0.34 |
| G*MA*-1B | 17.33 | 22.53 | 19.02 | 23.93 | Leipzig-Holzhausen | 51°19'2"N | 12°24'45"E | 149 | 18.61 ± 0.29 | 24.11 ± 0.34 |
| G*MA*-2 | 17.33 | 22.53 | 19.02 | 23.93 | Leipzig-Holzhausen | 51°19'2"N | 12°24'45"E | 150 | 18.61 ± 0.29 | 24.11 ± 0.34 |
| G*MA*-3 | 17.33 | 22.53 | 19.02 | 23.93 | Leipzig-Holzhausen | 51°19'2"N | 12°24'45"E | 151 | 18.61 ± 0.29 | 24.11 ± 0.34 |
| G*RK*-1 | 17.45 | 22.53 | 19.20 | 24.07 | Kubschütz/Bautzen | 51°9'42"N | 14°30'20"E | 232 | 18.43 ± 0.28 | 23.93 ± 0.34 |
| I*T*-1 | 20.43 | 26.53 | 22.68 | 28.87 | Trentino Roncafort | 46°5’44”N | 11°6’5”E | 194 | 22.00 ± 0.31 | 28.27 ± 0.39 |
|  |  |  |  |  | Trentino Laste | 46°4’19”N | 11°8’8”E | 312 |  |  |
| F*VC*-2A | 20.42 | 25.23 | 22.73 | 27.67 | Le-Luc | 43°22'48"N | 6°22'48"E | 80 | 24.05 ± 0.27 | 31.34 ± 0.32 |
| GR*L*-1 | 17.07 | 23.80 | 19.87 | 26.93 | Berovo (Macedonia) | 41°43'0"N | 22°51'0"E | 836 | 19.38 ± 0.25 | 25.90 ± 0.37 |
| PO*E*-1 | 24.55 | 31.97 | 27.13 | 34.97 | Badajoz (Spain) | 38°52'48"N | 6°48'36"W | 185 | 26.33 ± 0.25 | 34.44 ± 0.29 |
| ES*H*-2 | 23.72 | 30.60 | 26.70 | 33.90 | Hellin | 38°29'15"N | 1°42'45"W | 579 | 24.06 ± 0.26 | 32.71 ± 0.32 |
| IN*P*-3 | 27.20 | 31.40 | 28.55 | 32.80 | Palu | 0°40'48"S | 119°43'48"E | 6 | 28.06 ± 0.08 | 34.32 ± 0.11 |
| IN*K*-1 | 17.72 | 22.23 | 19.42 | 24.30 | -- | -- | -- | -- | -- | -- |
| IN*L*-1 | 21.42 | 25.77 | 23.05 | 27.73 | -- | -- | -- | -- | -- | -- |

Habitat mean surface air temperature (*T*_hab, mean_) and averaged maximum surface air temperature (*T*_hab, max_) during the warmest three months (in °C) were derived from the WorldClim database, using the CCM3 climate change model and nearby meteorological station data. Standard errors are reported for the meteorological station data only, because data from the WorldClim database did not allow for error estimation.
