# Supplementary material for: Coping with Temperature at the Warm Edge – Patterns of Thermal Adaptation in the Microbial Eukaryote Paramecium caudatum
Source: PLoS One. 2012 Mar 9;7(3):e30598. doi: 10.1371/journal.pone.0030598 (PMC3302864; doi:10.1371/journal.pone.0030598)
Supplement: Table S3 — Mantel test for the correlation between geographic ( x matrix) and ecophysiological distances ( y matrix). (DOC) [file pone.0030598.s003.doc]

**Table S3.** Mantel test for the correlation between geographic (*x* matrix) and ecophysiological distances (*y* matrix)

| ***y* matrix** | | ***SSx*** | ***SSy*** | ***SPxy*** | ***Rxy*** | ***p*-value** |
| --- | --- | --- | --- | --- | --- | --- |
| whole dataset (n = 18) | |  |  |  |  |  |
|  | C*T*_min_ | 3515845077.1 | 1150.4 | 1543819.3 | 0.768 | 0.001 |
|  | *T*_opt_ | 3515845077.1 | 72.6 | 377860.3 | 0.748 | 0.001 |
|  | C*T*_max_ | 3515845077.1 | 141.4 | 443627.1 | 0.629 | 0.001 |
| European subset (n =15) | |  |  |  |  |  |
|  | C*T*_min_ | 63391241.8 | 341.4 | 45438.8 | 0.309 | 0.086 |
|  | *T*_opt_ | 63391241.8 | 11.0 | 7186.4 | 0.272 | 0.092 |
|  | C*T*_max_ | 63391241.8 | 46.2 | 12596.5 | 0.233 | 0.096 |

*SSx* = sum of products of *x* matrix elements;

*SSy* = sum of products of *y* matrix elements;

*SPxy* = sum of cross products of corresponding elements of the *x* and *y* matrices;

*Rxy* = Mantel correlation coefficient
