# Supplementary material for: Coping with Temperature at the Warm Edge – Patterns of Thermal Adaptation in the Microbial Eukaryote Paramecium caudatum
Source: PLoS One. 2012 Mar 9;7(3):e30598. doi: 10.1371/journal.pone.0030598 (PMC3302864; doi:10.1371/journal.pone.0030598)
Supplement: Table S4 — Mantel test for the correlation between genetic ( x matrix) and geographic distances ( y matrix). (DOC) [file pone.0030598.s004.doc]

**Table S4.** Mantel test for the correlation between genetic (*x* matrix) and geographic distances (*y* matrix)

| **Source of data** | ***SSx*** | ***SSy*** | ***SPxy*** | ***Rxy*** | ***p*-value** |
| --- | --- | --- | --- | --- | --- |
| whole dataset (n = 18) | 111932.2 | 3515845077.1 | 19566361.8 | 0.986 | 0.001 |
| European subset (n = 15) | 1240.8 | 63391241.8 | 138057.5 | 0.492 | 0.013 |

*SSx* = sum of products of *x* matrix elements;

*SSy* = sum of products of *y* matrix elements;

*SPxy* = sum of cross products of corresponding elements of the *x* and *y* matrices;

*Rxy* = Mantel correlation coefficient
